# Biochemical and Molecular Characterization of Glycerol Dehydrogenase from *Klebsiella pneumoniae*

**DOI:** 10.4014/jmb.1909.09056

**Published:** 2019-10-22

**Authors:** Gyeong Soo Ko, Quyet Thang Nguyen, Do Hyeon Kim, Jin Kuk Yang

**Affiliations:** 1 Department of Chemistry, College of Natural Sciences, Soongsil University, Seoul 06978, Republic of Korea; 2 Department of Information Communication, Materials, and Chemistry Convergence Technology, Soongsil University, Seoul 06978, Republic of Korea

**Keywords:** Glycerol dehydrogenase, *gldA*, dihydroxyacetone production

## Abstract

Glycerol dehydrogenase (GlyDH) catalyzes the oxidation of glycerol to dihydroxyacetone (DHA), which is the first step in the glycerol metabolism pathway. GlyDH has attracted great interest for its potential industrial applications, since DHA is a precursor for the synthesis of many commercially valuable chemicals and various drugs. In this study, GlyDH from *Klebsiella pneumoniae* (KpGlyDH) was overexpressed in *E. coli* and purified to homogeneity for biochemical and molecular characterization. KpGlyDH exhibits an exclusive preference for NAD^+^ over NADP^+^. The enzymatic activity of KpGlyDH is maximal at pH 8.6 and pH 10.0. Of the three common polyol substrates, KpGlyDH showed the highest *k_cat_*/*K_m_* value for glycerol, which is three times higher than for racemic 2,3-butanediol and 32 times higher than for ethylene glycol. The *k_cat_* value for glycerol oxidation is notably high at 87.1 ± 11.3 sec^-1^. KpGlyDH was shown to exist in an equilibrium between two different oligomeric states, octamer and hexadecamer, by size-exclusion chromatography analysis. KpGlyDH is structurally thermostable, with a T_m_ of 83.4°C, in thermal denaturation experiment using circular dichroism spectroscopy. The biochemical and biophysical characteristics of KpGlyDH revealed in this study should provide the basis for future research on its glycerol metabolism and possible use in industrial applications.

## Introduction

**Fig. 1:**
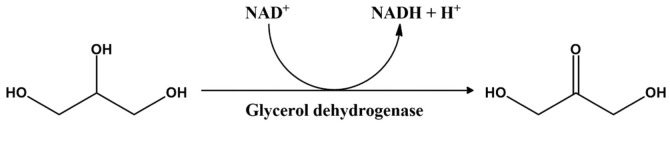
Glycerol oxidation catalyzed by glycerol dehydrogenase. NAD^+^ is the specific cofactor accepting the hydride ion resulting from the oxidation of glycerol.

Glycerol dehydrogenase (GlyDH, E.C.:1.1.1.6) catalyzes the oxidation of glycerol to dihydroxyacetone (DHA) by using NAD^+^ as an electron-accepting cofactor (Fig. 1) [1]. This reaction is the first step of the glycerol metabolism pathway in diverse prokaryotic organisms, a reaction that utilizes glycerol as a carbon source. Moreover, GlyDH is responsible, at least partly, for the conversion of acetoin to 2,3-butanediol in vivo [2]. In addition to glycerol and 2,3-butanediol, GlyDH was shown to catalyze the dehydrogenation of several other poly-hydroxyl chemical species (polyols), suggesting that this enzyme has a broad specificity toward polyol substrates. GlyDH is not only important in metabolic reactions but is also a valuable enzyme for industrial purposes. For example, the DHA produced by GlyDH is a precursor for a wide range of chemical products, from antifreeze fluids, such as 1,2-propylene glycol, to numerous biologically active compounds including drugs, pesticides, and sweeteners [3-5]. In addition, GlyDH is indispensable in the metabolic pathways involved in the production of biofuels such as ethanol, 1,3-propanediol and 2,3-butanediol [6-9]. As such, due to the fact that glycerol is the major byproduct of the biofuel production process, research regarding glycerol metabolism has increased in recent years, in order to increase the viability of the biofuel industry [2].

*Klebsiella pneumoniae* has been known to be one of the most efficient bacteria in glycerol utilization, which may be due to the fact that it carries two genes that encode GlyDH (*dhaD* and *gldA*). In order to use *K. pneumoniae* for industrial applications, namely for DHA production and the metabolization of the glycerol resulting from the biofuel production process, it is necessary to investigate the biochemical and biophysical characteristics of *K. pneumoniae*-derived GlyDH. In this study, GlyDH encoded by the *gldA* gene from the *K. pneumoniae* strain MGH78578 (KpGlyDH, hereafter), was recombinantly produced and purified, as it showed higher catalytic efficiency than the GlyDH encoded by the *dhaD* gene [2]. Subsequently, we carried out molecular analyses of size-exclusion chromatography, circular dichroism, and thermal denaturation. In addition, we investigated its enzymatic characteristics, such as optimal pH and temperature, and steady-state kinetic features toward several polyol substrates including glycerol.

## Materials and Methods

### Chemicals, Bacterial Strains, and Culture Conditions

All reagents were purchased from Sigma-Aldrich (USA) and BioShop (Canada). Genomic DNA from the *K. pneumoniae* strain MGH78578 (ATCC 700721) was purchased from ATCC (USA). A PCR kit and mini plasmid kit from GeneAll (Korea) were used for DNA purification and plasmid isolation. The *E. coli* strain DH10B was used for cloning, while Rosetta2(DE3) cells were used for gene expression. Cells were cultured in LB broth containing 30 μg/ml kanamycin at 37°C.

### Accession Numbers for Nucleotide Sequence and Amino Acid Sequence

DNA information was retrieved from the NCBI GenBank database (https://www.ncbi.nlm.nih.gov/genbank/). The gene ID of *gldA* reported in this paper is ABR79620, and the genome accession number of *K.pneumoniae* MGH78578 is CP000647. The protein information was retrieved from the UniProt database (https://www.uniprot.org/uniprot/A6TGD6) with the accession number being A6TGD6.

### Construction of Expression Plasmid

The KpGlyDH-encoding gene, *gldA*, was amplified using the genomic DNA of the *K. pneumoniae* strain MGH78578 as a template. The primer sequences are as follows, 5‘-AGCGACGCATATGGATCGCATTATTCAA-3’(forward) 5’-CTGTTCTCGAGTTCCCACTCTTGAAGGAAGCGCTGACC-3’ (reverse).

The underlined sequences represent NdeI and XhoI restriction sites.

Subsequently, the amplified PCR product was digested using the NdeI and XhoI digestion enzymes and then ligated into the pET26b vector (Novagen, USA), which added a His6-tag to the Cterminus of the protein encoded by the inserted gene. The ligated vector was then transformed into *E. coli* DH10B cells and colonies were selected by culturing transformed cells on LB plates containing 30 μg/ml kanamycin. The nucleotide sequences were confirmed by sequencing (Bionics, Korea). All subsequent DNA manipulation proceeded according to standard protocols.

### Expression and Purification of Recombinant KpGlyDH

The constructed *gldA* expression plasmid was transformed into Rosetta2(DE3) cells, which were cultured in LB broth at 37°C. Subsequently, IPTG (isopropyl-β-D-1-thiogalactopyranoside) was added at OD600 = 0.5-0.8 and a final concentration of 0.5 mM. The cells were then further cultured at 37°C for 4 h and then at 20°C overnight. The cultured cells were harvested by centrifugation (Hanil Combi-541R; 3000 rpm, 15 min), and cell pellets were resuspended in a working buffer containing 20 mM Tris-HCl, 0.1 mM TCEP, 5% ethylene glycol, 100 mM NaCl, and 10 mM imidazole, pH 8.0. The cells were lysed by sonication and the supernatant was used for protein isolation, by using different purification columns in the following sequence: HisTrapFF, HiPrep 26/10 Desalting, and HiLoad 16/600 Superdex-200 (GE Healthcare, USA). The protein purity was checked by SDS-PAGE, and the concentration was measured using NanoDrop 1000 (Thermo Scientific, USA). The final purified protein was concentrated to 31.7 mg/ml and stored at -60°C for subsequent analyses.

### Activity Assay and Kinetic Analysis

GlyDH enzyme activity was assayed spectrophotometrically, by measuring the increase in NADH concentration at 35°C, at a wavelength of 340 nm, using a Ultrospec 8000 UV/Vis spectro-photometer (GE Healthcare). The enzyme reaction was monitored for 3 min following the start of the reaction, in a total reaction volume of 400 μl. Purified KpGlyDH in 20 mM HEPES, pH 7.4 was used. Each assay was performed three times independently.

The optimal pH for glycerol oxidation was investigated using several different buffer system at 200 mM concentration from pH 6.0 to pH 11.0; ADA buffer (pH 6.0 and 6.5) and HEPES buffer (pH 7.0, 7.5, and 8.0), CHES buffer (pH 8.6, 9.0, and 9.5), and CAPS (pH 10.0, 10.5, and 11.0) at 35°C. To compare the enzyme activity in two different buffers of Tris and CHES, the reaction was monitored at pH 8.6 buffered by 200 mM Tris and CHES separately. In order to determine cofactor specificity toward NAD^+^ and NADP^+^, 10 mM NAD^+^ or NADP^+^ were added to the reaction mixture containing 10 nM KpGlyDH, 1.0 M glycerol, and 200 mM CHES, pH 8.6. To investigate the temperature dependence of the enzyme activity, the enzyme activity was measured at various temperatures from 25°C to 60°C with an increment of 5°C in a reaction mixture containing 10 nM KpGlyDH, 1.0 M glycerol, 10 mM NAD^+^ and 200 mM CHES buffer, pH 8.6.

In order to determine the kinetic parameters for three different polyol substrates (glycerol, ethylene glycol, and 2,3-butanediol racemate), as well as for the specific cofactor NAD^+^, the increase in NADH concentration was spectrophotometrically quantitated, by measuring the increase in absorbance at 340 nm at 35°C. The reaction mixture consists of 10 nM KpGlyDH, 10 mM NAD^+^, 200 mM CHES buffer, pH 8.6, and the polyol substrate. The initial reaction rate was measured for glycerol, ethylene glycol, and 2,3-butanediol racemate at seven different concentrations, ranging from 0.2 mM to 800 mM, 10 mM to 2,000 mM, and 1 mM to 1,000 mM, respectively. In order to determine the kinetic parameters for the specific cofactor NAD^+^, it was added in concentrations ranging from 0.1 mM to 10 mM in the reaction mixture containing 10 nM KpGlyDH, 1.0 M Glycerol, and 200 mM CHES buffer, pH 8.6. All apparent kinetic parameters were calculated by plotting the data, using Origin 9.0 (Origin Lab).

### Analytical Size-Exclusion Chromatography

Size-exclusion chromatography analysis was performed on the AKTA basic system (GE Healthcare). Superdex-200 10/300 GL (GE life science, USA) was pre-equilibrated with the working buffer (20 mM Tris-HCl, 0.1 mM TCEP, and 200 mM NaCl, pH 8.0). The injected protein sample of 200 μl was run through the column at a flow rate of 0.5 ml/min. Blue Dextran (Sigma-Aldrich, D4772) was used to determine void volume. The standard mix used was composed of eight proteins: aprotinin from bovine lung (Sigma-Aldrich, A3886), cytochrome C from equine heart (Sigma-Aldrich, C7150), carbonic anhydrase from bovine erythrocytes (Sigma-Aldrich, C7025), ovalbumin from chicken egg white (Sigma-Aldrich, A8531), alcohol dehydrogenase from yeast (Sigma-Aldrich, A8656), β-amylase from sweet potato (Sigma-Aldrich, A8781), and apoferritin from horse spleen (Sigma-Aldrich, A3630).

### Circular Dichroism (CD) Spectroscopy and Thermal Denaturation Analysis

The far-UV CD spectra of the purified KpGlyDH (0.23 mg/ml) enzyme was recorded in a solution containing 20 mM Tris–HCl, 200 mM NaCl, pH 7.5 at 20°C, using a 0.4 cm path-length cell on a Jasco J-710 spectropolarimeter (JASCO, USA) equipped with a temperature controller. The CD spectra were recorded for three individual scans from 190 nm to 260 nm (0.1 nm step resolution, 1 nm bandwidth, and 1 s response time). The three spectra were summed and averaged, followed by the subtraction of the solvent CD signal. The CD intensity at a wavelength λ was normalized as the mean residue molar ellipticity. Thermal denaturation experiments were carried out at a wavelength of 222 nm, using protein samples with a concentration of 0.45 mg/ml. The CD intensity was recorded every 30 sec as the temperature increased from 25°C to 95°C, at a speed of 2°C/min.

## Results

### KpGlyDH Expression and Purification

**Fig. 2:**
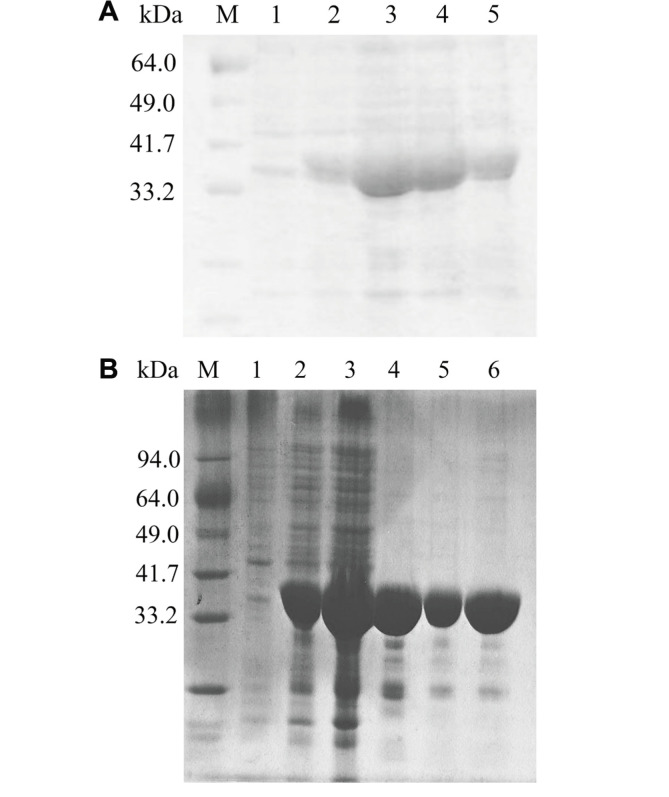
KpGlyDH overexpression and purification. (**A**) Small scale expression test for the recombinant KpGlyDH. Cterminally His-tagged KpGlyDH expression was tested in *E. coli* and Rosetta2(DE3) system at 20°C. Lane M: protein marker. Lane 1: before IPTG addition. Lanes 2 and 3: three hours and overnight after IPTG addition. Lanes 4 and 5: supernatant and precipitation after cell lysis. (**B**) Purification of recombinant KpGlyDH. Lane M: protein marker. Lane 1: before IPTG addition. Lane 2: overnight, after IPTG addition. Lane 3: supernatant after cell lysis. Lane 4: eluted from HisTrapFF column. Lane 5: after Superdex-200 column. Lane 6: final concentrated sample.

In the overexpression test culture, KpGlyDH was expressed mostly as inclusion bodies at 37°C and showed a tendency to increase in the soluble fraction as the culture temperature was lowered. At the culture temperature of 20°C, KpGlyDH was mostly expressed as a soluble protein (Fig. 2A). The band reflecting induced KpGlyDH expression emerged between the markers for 33.2 kDa and 41.7 kDa, which is consistent with its calculated mass of 39.8 kDa. The final purified sample showed more than 95%homogeneity, as shown by the SDS-PAGE (Fig. 2B).

### Enzymatic Characterization

**Fig. 3:**
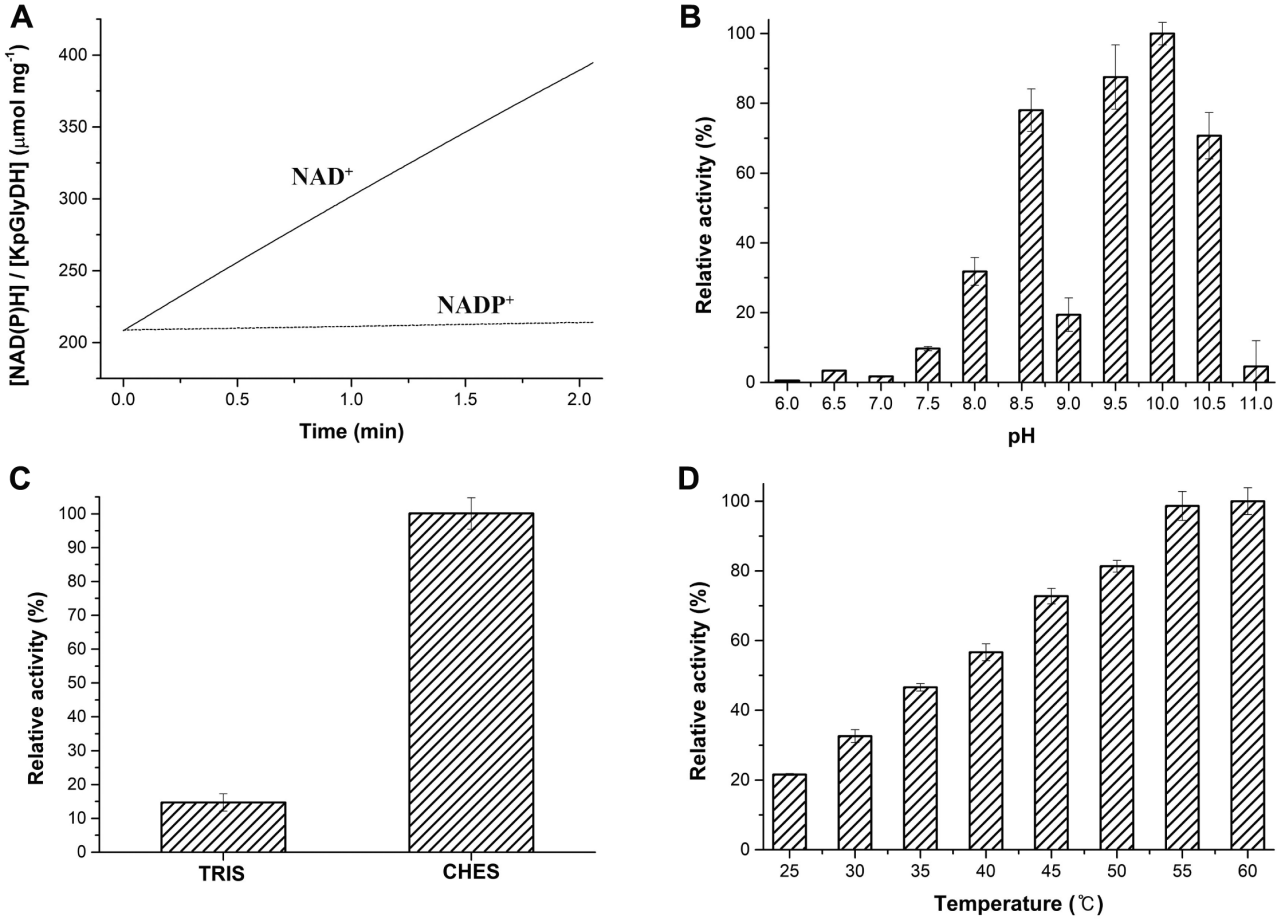
Optimum conditions for KpGlyDH. (**A**) Cofactor specificity. (**B**) pH dependence of the enzymatic activity. The activity at pH 10.0 is set to 100% for comparison. (**C**) Two buffer systems at pH 8.6. (**D**) Temperature dependence of the enzymatic activity.

In order to determine the cofactor specificity between NAD^+^ and NADP^+^, we assayed the rate of glycerol oxidation catalyzed by KpGlyDH for the two electron-accepting cofactors, at pH 8.6 and 35°C (Fig. 3A). The assay results clearly showed the definite preference for NAD^+^ with a specific activity of 101.68 ± 4.51 μmol min^-1^ mg^-1^, while the specific activity in the presence of NADP^+^ was 2.78 ± 0.05 μmol min^-1^ mg^-1^ (Fig. 3A).

The pH dependence of KpGlyDH activity was determined by measuring the specific activity for glycerol oxidation at eleven different pH points from 6.0 to 11.0 using several different buffer systems such as ADA, HEPES, CHES, and CAPS. The profile shows dual maximal at pH 8.6 of CHES buffer and pH 10.0 of CAPS buffer, with the global maximum at pH 10.0. If we consider the activity at pH 10.0 as 100%, then the activity at pH 8.6 is 78.0% (Fig. 3B). Even though the global maximum was pH 10.0, we chose pH 8.6 for all the enzyme assay experiments in this study since it is closer to the physiological condition than pH 10.0. Notably, GlyDH from *Thermoanaerobacterium thermosaccharolyticum* also showed the maximal activity for glycerol oxidation at pH 8.0, even though it was not investigated over pH 9.0 [10].

Intriguingly, we found that Tris buffer greatly lowers the enzyme activity in comparison to CHES buffer. When the specific activity was compared commonly at pH 8.6 for both buffer systems, the activity in CHES buffer is around 7-fold higher than in Tris buffer (Fig. 3C).

We also carried out the glycerol oxidation assay at different reaction temperatures from 25°C to 60°C to investigate the temperature dependence of the enzyme activity. As the temperature increases, the specific activity keeps increasing up to 60°C although the activity seems to reach almost the maximum from 55°C (Fig. 3D).

### Determination of the Apparent Kinetic Parameters

In order to determine kinetic parameters for the specific cofactor NAD^+^, Michaelis-Menten kinetics experiments were carried out for the glycerol oxidation at 35°C and pH 8.6. The *K_m_* for NAD^+^ was determined as 2.6 ± 0.6 mM, and *V_max_* was 106.4 ± 14.6 μmol min^-1^ mg^-1^ (Table 1). Subsequently, we determined the kinetic parameters for three different polyol substrates: glycerol, ethylene glycol, and 2,3- butanediol. The *K_m_* values for the three are comparable in overall: glycerol and ethylene glycol showed very similar *K_m_* values, 91.0 ± 23.8 mM and 92.7 ± 22.2 mM, respectively, and 2,3-butanediol racemate has slightly lower *K_m_* value of 67.3 ± 12.3 mM. However, *k_cat_* values, in contrast, are significantly different for the three substrates. Glycerol is the highest with 87.1 ± 11.3 sec^-1^, and 2,3-butanediol racemate and ethylene glycol are around 4-times and 30- times lower, respectively (Table 1). As a consequent, the specificity constant, *k_cat_*/*K_m_*, is the highest for glycerol (0.96 ± 0.00 sec^-1^ mM^-1^) among the three by significant differences. GlyDH’s from *Thermotoga maritima* and *Klebsiella pneumoniae* also showed the highest specificity toward glycerol [3].

### Size-Exclusion Chromatography Analysis

**Fig. 5:**
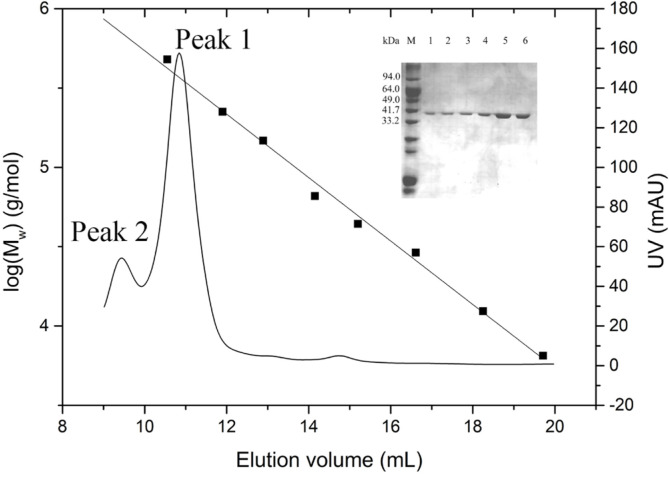
Size exclusion chromatography analysis of KpGlyDH. Eight different standard proteins are used for reference. Details are in *Methods and Materials*. Peak 1 eluted at 10.8 ml and Peak 2 at 9.5 ml. Lanes 1-3 in SDS-PAGE are for Peak 2 and lanes 4-6 for Peak 1.

In order to investigate the oligomeric state of KpGlyDH, we performed a size-exclusion chromatography analysis. The purified KpGlyDH (4.94 mg/ml) was run through an analytical Superdex-200 (GE Healthcare) column, and its elution volume, V_e_, was measured and compared with those of standard proteins (Fig. 5). Two well-separated peaks emerged at a V_e_ of 9.5 ml and 10.8 ml, respectively. The molecular mass values for each peak were calculated from the standard curve; ~710 kDa for Peak 2 and ~370 kDa for Peak 1 which are ~18 times and ~9 times, respectively, larger than the calculated molecular mass of 39.8 kDa for a single chain of His-tagged recombinant KpGlyDH. Considering that the octamer of GlyDHs from *Bacillus stearothermophilus* was observed both in solution and in the crystal lattice [11] and that the hexadecamer (dimer of the octamer unit) was observed in the crystal lattice of GlyDH from *Serratia plymuthica* [12], Peak 1 may be represented by the octamer of KpGlyDH and Peak 2 by the hexadecamer. The octamer of Peak 1 apparently dominates in quantity over the hexadecamer of Peak 2. As such, the equilibrium is leaning toward the lower mass oligomer, the octamer.

### Thermal Stability Investigated by Circular Dichroism Spectroscopy

**Fig. 6:**
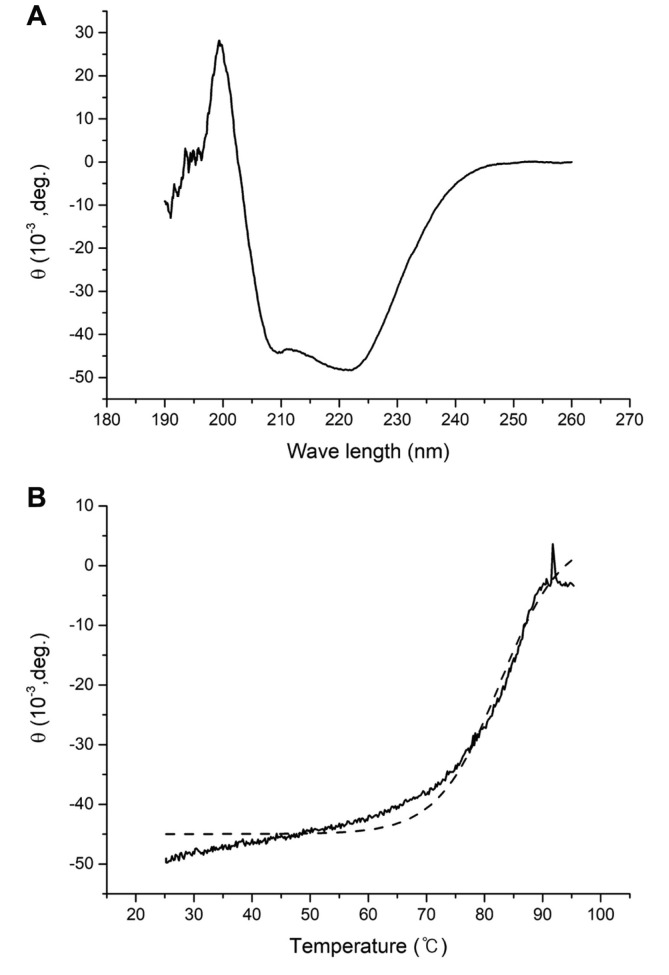
Circular dichroism analysis of KpGlyDH. (**A**) Wavelength scan. (**B**) Thermal denaturation at 222 nm. The solid line represents the raw measurements, while the dotted line represents the sigmoidal fitting.

The CD spectra were obtained by a wavelength scan from 190 nm to 260 nm at 25°C. The spectra exhibit the characteristic pattern for an α/β protein (Fig. 6A), which is in good agreement with the previously reported crystal structures of GlyDHs from other organisms containing the α/β Rossman fold domain [11-14]. Afterwards, the wavelength was fixed at 222 nm, and the temperature varied between 25°C and 95°C. A total of 348 data points were collected for the temperature range, and the data were subjected to sigmoidal fitting (Fig. 6B). The melting temperature (T_m_) for KpGlyDH was estimated as 83.4°C, which is relatively high, especially when considering the fact that *Klebsiella pneumoniae* is a mesophile.

## Discussion

In this study, we investigated molecular and enzymatic characters of the glycerol dehydrogenase encoded by the *gldA* gene of *K. pneumoniae* (KpGlyDH). Our results showed that glycerol is more specific substrate than 2,3-butanediol racemate and ethylene glycol, as judged by *k_cat_*/*K_m_*. The difference is mainly contributed by *k_cat_*, not *K_m_*, implying that the catalytic turnover potency is different while the binding affinity is similar. During the determination of pH dependence, we observed interesting phenomenon that Tris buffer has an inhibitory effect on the enzymatic activity of KpGlyDH. An even more intriguing observation for the pH dependence was the dual maxima of the activity at pH 8.6 and 10.0 with a sharp drop in between at pH 9.0 (Fig. 3B). This unusual profile of the pH dependence might be related, at least in part, to the reaction mechanism involving the proton transfer between the catalytic residues and the substrate in the active site, which could be inferred from the ongoing subsequent studies for its three-dimensional structure determination.

Notably, KpGlyDH in this study exhibits remarkably high *k_cat_* value of 87.1 ± 11.3 sec^-1^ which is about 5-times higher than the other study [2]. This may be attributed to the difference of the reaction condition, espeically to the higher temperature of our assay (35°C) than the other one (30°C).

As shown by analytical size-exclusion chromatography analysis of the purified KpGlyDH, it exists in two different oligomeric states in equilibrium: octamer and hexadecamer. In the previous studies on GlyDH from *Bacillus stearothermophilus*, only octamer is observed commonly in size-exclusion chromatography and in the crystal lattice (PDB code 1JQA) while only tetramer was seen in electron microscopy [11]. GlyDH from *T. maritima* also forms octamer in the crystal lattice (PDB code 1KQ3), even though its oligomeric state in solution was not investigated [14]. On the other hand, GlyDH from *Serratia plymuthica* forms only tetramer in solution as judged by size-exclusion chromatography [12]. So, to our knowledge, our study is the first time to observe that GlyDH forms hexadecamer in solution and also that GlyDH exists in both states of octamer and hexadecamer at the same time in equilibrium. These studies on the oligomeric assembly of GlyDH’s from four different species including *K. pneumoniae* of our study may suggest that the GlyDH can form a C4-symmetry tetramer as a basic oligomeric unit and assemble into a higher-order oligomer such as octamer and hexadecamer by repeating the tetramer unit depending on conditions presumably such as protein concentration, pH or ionic strength. With regards to hexadecamer assembly, the crystal structure of GlyDH from *Serratia plymuthica* seems to show a plausible assembly mode in the crystal lattice (PDB code 4MCA), even though it is not discussed in the report [12], in which an octamer is formed by 42-symmetry and then two octamers are stacked in a head-to-head fashion through a four-fold axis with a 90o angle difference.

The structure of KpGlyDH was very thermostable in the thermal denaturation with circular dichroism spectroscopy, so that its T_m_ was measured as 83.4°C. It is quite surprising especially considering that *K. pneumoniae* is a mesophile. When considering the well-established principle for the correlation between thermostability and oligomeric assembly, the unusually high T_m_ for KpGlyDH could be attributed, at least in part, to its high level of oligomeric assembly to the octamer or the hexadecamer which were observed through the size-exclusion chromatography (Fig. 5). In addition to the structural thermostability, the glycerol oxidation rate increases as the reaction temperature is raised up to 60°C (Fig. 3D). So, it is plausible to explain the faster reaction rate at the higher temperature as the result of the resistance to the thermal denaturation at least for the reaction time of three minutes. Considering the potential applications of GlyDH for the industrial production of DHA from glycerol or for the metabolization of co-produced glycerol in the biodiesel production process [6-9], the outstanding structural stability of KpGlyDH may add to its industrial value. From this perspective, the relatively very high turnover number of KpGlyDH for glycerol oxidation, 87.1 ± 11.3 sec^-1^, even at 35°C is also worth to note, especially with consideration that KpGlyDH showed in this study the faster glycerol oxidation rate at higher temperature as mentioned above. Conclusively, the molecular and enzymatic characteristics of KpGlyDH were investigated in this study, and the current findings lay the groundwork for future studies on the glycerol metabolism and the attempts for its possible use in industrial applications.

## Figures and Tables

**Fig. 4:**
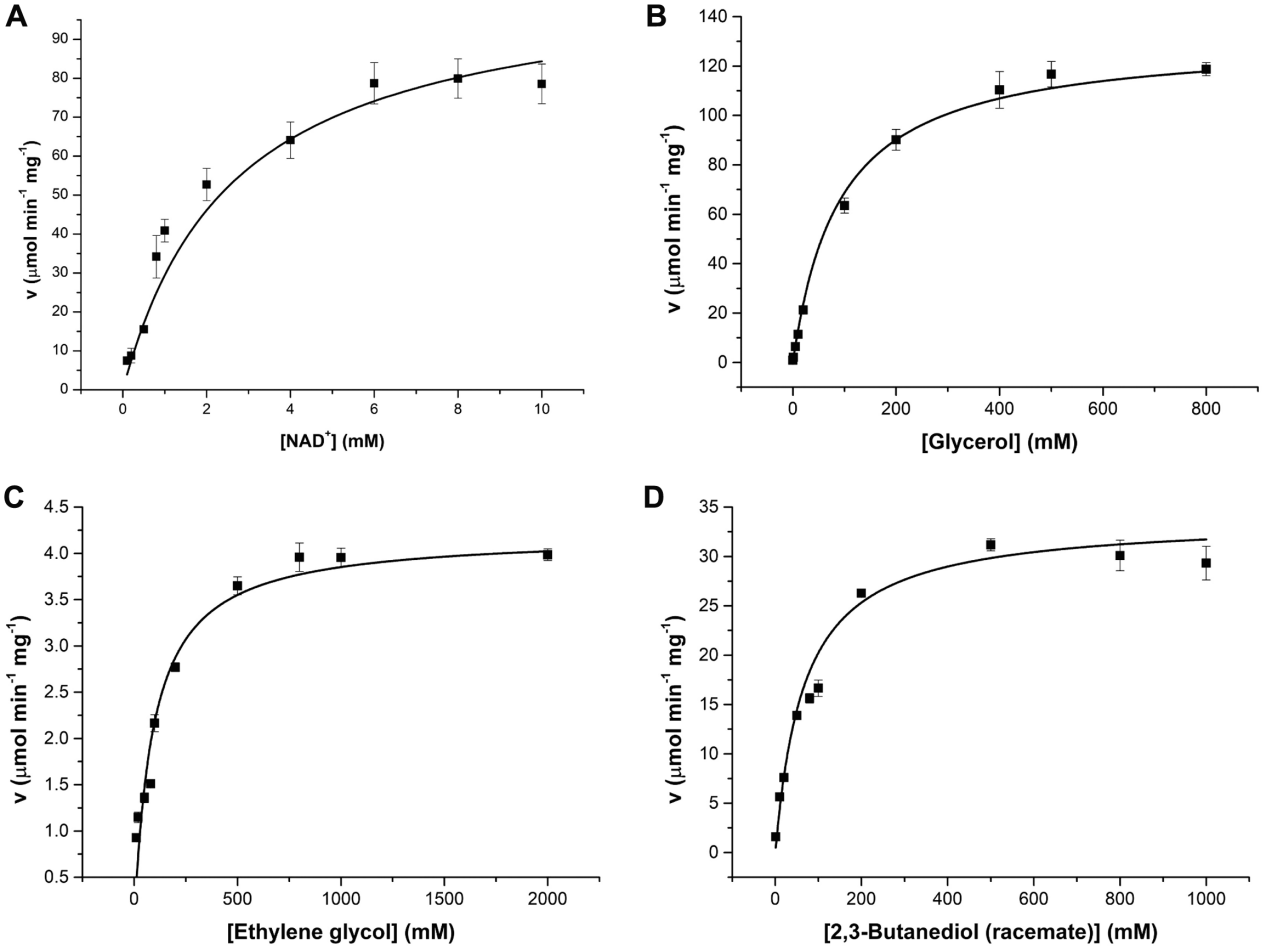
Michaelis-Menten kinetics of KpGlyDH. (**A**) Reduction of cofactor NAD^+^ at varying concentrations, with glycerol as substrate. (**B**) - (**D**) Oxidation of polyol substrates: glycerol, ethylene glycol, and 2,3-butanediol racemate.

**Table 1:** Kinetic parameters for three different polyol substrates and the cofactor.

Substrate	*V_max_* (μmol min^-1^ mg^-1^)	*K_m_* (mM)	*k_cat_* (sec^-1^)	*k_cat_*/*K_m_* (sec^-1^ mM^-1^)
Glycerol	131.2 ± 17.1	91.0 ± 23.8	87.1 ± 11.3	0.96 ± 0.00
Ethylene glycol	4.2 ± 0.3	92.7 ± 22.2	2.8 ± 0.2	0.03 ± 0.00
2,3-butanediol racemate	33.9 ± 2.5	67.3 ± 12.3	22.5 ± 1.7	0.33 ± 0.00
NAD^+^	106.4 ± 14.6	2.6 ± 0.6	70.6 ± 9.7	27.0 ± 0.2
